# Comprehensive left ventricular mechanics analysis by speckle tracking echocardiography in Chagas disease

**DOI:** 10.1186/s12947-016-0062-7

**Published:** 2016-05-27

**Authors:** Marcio Silva Miguel Lima, Hector R. Villarraga, Maria Cristina Donadio Abduch, Marta Fernandes Lima, Cecilia Beatriz Bittencourt Viana Cruz, Marcio Sommer Bittencourt, Mariana Callil Voos, Joao Cesar Nunes Sbano, Wilson Mathias, Jeane Mike Tsutsui

**Affiliations:** 1 Serviço de Ecocardiografia, Instituto do Coração (InCor), University of Sao Paulo Medical School, Av Dr Eneas de Carvalho Aguiar, 44, Cerqueira Cesar, 05.403-000 Sao Paulo, SP Brazil; 2 Mayo Clinic and Mayo Foundation, Rochester, MN USA

**Keywords:** Speckle tracking echocardiography, Cardiomyopathies, Cardiac mechanics, Strain

## Abstract

**Background:**

Chagas disease (CD) is a frequent cause of dilated cardiomyopathy (CMP) in developing countries, leading to clinical heart failure and worse prognosis. Therefore, the development and evolution of this CMP has always been a major topic in numbers of previous studies. A comprehensive echocardiographic study of left ventricular (LV) mechanics, fully assessing myocardial contraction, has never been done before. This could help characterize and improve the understanding of the evolution of this prevalent CMP.

**Methods:**

A total of 47 chagasic and 84 control patients were included in this study and allocated in groups according to LV ejection fraction. 2D-Echocardiogram was acquired for LV mechanics analysis by speckle tracking echocardiography.

**Results:**

Mean age of chagasic individuals was 55y and 16 (34 %) were men. Significant difference was found in global longitudinal velocity analysis, with lower values in indeterminate form. In the group with severe systolic dysfunction, a paradoxical increase in longitudinal and apical radial displacements were demonstrated. In parallel, segmental analyzes highlighted lower values of radial displacement, strain and strain rate into inferior and inferolateral walls, with increase of these values in septal and anterior walls.

**Conclusion:**

Chagasic CMP has a vicarious pattern of contraction in the course of its evolution, defined by reduced displacement and strain into inferior and posterior walls with paradoxical increase in septal and anterior segments. Also, lower longitudinal velocities were demonstrated in CD indeterminate form, which may indicate an incipient myocardial injury.

## Background

Chagas disease (CD), caused by the parasite *Trypanosoma cruzi*, is a serious health problem in Latin America and recently has also been emerging in non-endemic countries [1]. Dilated cardiomyopathy is the most important and severe manifestation of human chronic CD [2]. An identification of markers highlighting a progression of chagasic cardiomyopathy (CMP) is relevant for appropriate patient management. The most important predictors of death are limitation to activities (New York Heart Association functional class), left ventricular (LV) systolic dysfunction and non-sustained ventricular tachycardia, which reflect the severity of myocardial damage [3, 4]. Echocardiography has been used as an important tool in the assessment of cardiac abnormalities in this group of patients [5–8].

**Fig. 1:**
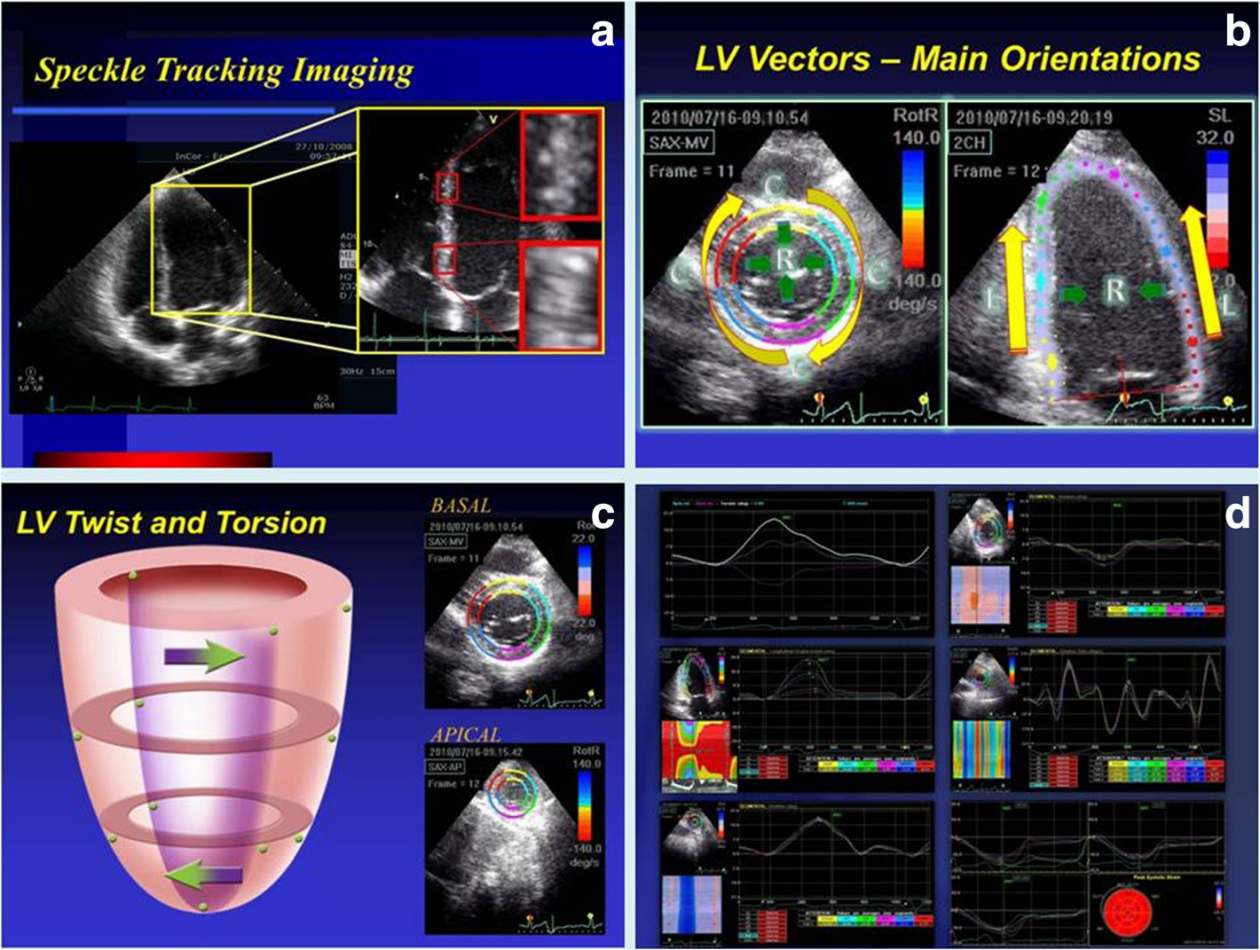
Speckle tracking echocardiography. Zoom image of myocardial ultrassonographic appearance, with the speckles (**a**). Main orientations of LV contration: R, radial; C, circumferential; L, longitudinal (**b**). Representation of LV twist – clockwise rotation at the basis and counterclockwise at the apex (**c**). Example of myocardial mechanics analysis (**d**)

There are few reports using other techniques besides left ventricular ejection fraction (LVEF) for chagasic patients clinical evaluation [9–12]. Mechanisms underlying pathways to LV dysfunction are poorly understood and, also, early stages of dysfunction may be enhanced by the assessment of myocardial contraction mechanics using speckle tracking echocardiography (STE) [13–22]. This new echocardiographic tool is based on identification and a frame-by-frame tracking of natural acoustic myocardial markers which arise from interaction of ultrasound wave and small myocardial elements. These speckles patterns are unique in each segment and their change in position allows determination of multiples parameters which comprises cardiac mechanics, such as LV strain and torsion (Fig. 1). Although there is a robust literature using general echocardiography in CD, a comprehensive echocardiographic study of LV mechanics, using this relative novel tool (STE), fully assessing myocardial contraction and describing CD evolution, has never been done before. This could help characterize and improve the understanding of the evolution of this prevalent CMP.

In this study we sought to compare the dynamics of LV contraction, through the determination of multiple echocardiographic parameters obtained by STE (such as strain, strain rate (SR), segmental rotation, rotational velocity, twist/torsion) between patients with CD and control group, with and without LV systolic dysfunction.

## Methods

### Study participants

This is a single center prospective study. From January 2010 to August 2013, we studied 135 patients with CD and controls. CD was confirmed by at least two positive serologic tests for *Trypanosoma cruzi* and patients were enrolled into 4 groups according to LVEF: **Ch1A,** normal LVEF (≥55 %) and normal 12-lead electrocardiogram (ECG); **Ch1B,** normal LVEF (≥55 %) and ECG abnormalities suggestive of myocardial involvement by CD, such as atrioventricular block, branch or fascicular block; **Ch2,** mild to moderate LV dysfunction (LVEF 54–31 %); **Ch3,** severe LV dysfunction (LVEF ≤ 30 %). We also included control patients without CD classified into 3 groups according to LVEF (≥55 %, 54–31 % and ≤ 30 %). Exclusion criteria were the presence of supraventricular arrhythmias (atrial fibrillation or flutter), systemic blood pressure over 180/110 mmHg, history of myocardial infarction or coronary artery disease, pacemaker, significant thyroid disease and end-stage renal failure.

The institutional review board approved the study (Heart Institute of Sao Paulo Medical School (InCor)), and all participants gave informed consent. All clinical investigations were conducted according to the principles expressed in the Declaration of Helsinki.

### Echocardiography and STE imaging

Echocardiography was performed on commercially available echocardiographic platforms equipped with MS5 probe (GE Vivid 7 and E9, GE Healthcare, Milwaukee, Wis). Comprehensive 2D-Echocardiogram and Doppler evaluation was performed following the recommendations of the American Society of Echocardiography [23]. LVEF was measured by Simpson’s rule. Diastolic function was evaluated by mitral inflow E/A pattern and annular tissue Doppler curves (e’/a’). Valves were assessed by color, pulsed and continuous Doppler. Pulmonary artery systolic pressure was estimated through the maximal velocity of obtained from tricuspid regurgitation jet.

The echo-STE protocol included acquisition of short axis and apical views. Parasternal short-axis views were obtained at the LV base (mitral valve level), at LV medium portion (papillary muscles level), and at the LV apex, close to apical obliteration when there is still a clear visualization of segments. For this apical “cut”, in order to avoid quantification bias, we created another new criterion: an identification of at least a tendency to rotation of the apex. This rotation could be either in a counterclockwise direction, physiologically expected, but also in a clockwise direction, as observed in some patients with significant LV systolic dysfunction.

Left ventricular twist is the wringing motion of heart around its long axis. It is calculated as the net absolute difference between apical and basal rotations (LV_twist_ = ROT_apical_ - ROT_basal_). Torsion is a normalization of LV twist to the length of LV long axis (LV_twist_/LV_lenth_). By widely assumed convention, apical rotation had positive values and basal, negative [24].

Acquisition of apical views (A3C, A2C and A4C) followed transversal images. Images were acquired at a frame-rate of 40–80 fps. Three consecutive heart cycles were stored.

Speckle tracking analysis was performed offline using a dedicated software (EchoPAC, v. BT10, GE Heathcare). For short axis images 7–12 and for apical 3 anchor points were placed. The software automatically defined the region of interest (ROI) for the entire myocardial layer, which was divided in six color-coded segments (total: 18 segments). Careful attention was especially given to not include myocardial trabecullaes and the pericardium. Adjustments were possible. Following this step, an automatic tracking of myocardial speckles were performed and final results on the quality of this tracking were given for each color-coded segment. If there was a suboptimal tracking of one segment, adjustment was also possible. After accepting this analysis, curves were given for all variables studied and this data exported to a spreadsheet. Global values were defined as the average of segments analyzed.

### Statistical analysis

Continuous variables are presented as mean ± standard deviation (SD) and categorical variables as numbers and proportions. Comparisons of continuous variables were performed by Student’s *t* test or analysis of variance (ANOVA) with post hoc Tukey HSD test. For categorical variables we used the chi-square test (*χ* ^2^) or Fisher’s exact test when appropriate. To test the effect of covariates of interest, an analysis of covariance (ANCOVA) followed by Bonferroni post hoc test and multiple linear regression was performed. Analysis of interobserver and intraobserver variability were performed on six randomized patients, three with normal LVEF and three with systolic dysfunction. A value of *p* < 0.05 was considered statistically significant, and a confidence interval of 95 %. Statistical analysis was performed using SPSS 20.0 for Macintosh (SPSS Inc., Chicago, IL).

## Results

### Baseline characteristics

Among the 135 initially enrolled patients, 4 were excluded due to poor acoustic resolution (2 from CD group and 2 from control group). The final study population was composed of 131 subjects, with the following distribution: Ch1A: 8 (6 %), Ch1B: 13 (10 %), Ch2: 17 (13 %) and Ch3: 9 (7 %); C1: 58 (44 %), C2: 7 (5 %) e C3: 19 (15 %) patients. The overall feasibility for STE analysis was 97 %. Mean age of chagasic individuals was 55 years-old and 16 (34 %) were men, whereas in controls mean age was 45 years-old, 38 (45 %) men. There were few patients with hypertension (mild stage) in the chagasic groups Ch1 and Ch2. The majority of patients of the entire population was in stage I of functional symptomatic classification. Heart rate of Ch2 group was higher than its control. No other significant differences in hemodynamic parameters were observed among groups. Clinical characteristics of CD and control groups are described in Table 1. Table 2 comprises cardiac variables obtained by conventional echocardiography. Worth to note was that some chagasic patients with normal cardiac function (Ch1 and Ch2) had findings of mild diastolic dysfunction comparing to controls.

**Table 1:** Clinical and demographic characteristics of groups

Variables	Chagas				Controls		
	Ch1A (*n* = 8)	Ch1B (*n* = 13)	Ch2 (*n* = 17)	Ch3 (*n* = 9)	C1 (*n* = 58)	C2 (*n* = 7)	C3 (*n* = 19)
Age (y)^a^	56 ± 7^c^	54 ± 12^c^	56 ± 12	56 ± 9	37 ± 12	47 ± 9	50 ± 11
Gender M^b^	1 (12 %)	4 (31 %)	8 (47 %)	3 (67 %)	25 (43 %)	3 (43 %)	10 (53 %)
Weight (Kg)^a^	66.3 ± 10.6	67.5 ± 12.5	68.3 ± 12.3	65.2 ± 8.6	73.1 ± 15.9	69.5 ± 14.6	69.8 ± 16.2
Height (cm)^a^	157 ± 12^d^	160 ± 10^d^	165 ± 8	162 ± 6	169 ± 9	162 ± 10	164 ± 12
BS (m^2^)^a^	1.66 ± 0.19	1.70 ± 0.18	1.64 ± 0.18	1.70 ± 0.12	1.83 ± 0.21	1.74 ± 0.21	1.75 ± 0.25
BMI (kg/m^2^)^a^	26.9 ± 3.5	26.6 ± 4.8	25.0 ± 3.0	24.8 ± 3.0	25.4 ± 4.1	26.2 ± 4.4	25.6 ± 3.9
SAH^b^	4 (50.0 %)^e^	5 (38.5 %)^e^	5 (29.4 %)	4 (44.4 %)	0 (0 %)	1 (14.3 %)	8 (42.1 %)
DM^b^	1 (12.5 %)	1 (7.7 %)	1 (5.9 %)	1 (11.1 %)	0 (0 %)	1 (14.3 %)	1 (5.3 %)
DLP^b^	2 (25.0 %)^f^	4 (30.8 %)^f^	4 (23.5 %)	4 (44.4 %)	1 (1.7 %)	1 (14.3 %)	4 (21.1 %)
Smoker^b^	0 (0 %)	0 (0 %)	0 (0 %)	1 (11.1 %)	0 (0 %)	0 (0 %)	0 (0 %)
FC CHF (NYHA)^b^							
I	1 (12.5 %)^g^	7 (53.8 %)^g^	11 (64.7 %)	5 (55.5 %)	0 (0 %)	3 (42.9 %)	11 (57.9 %)
II	0 (0 %)	1 (7.7 %)	5 (29.4 %)	4 (44.4 %)	0 (0 %)	3 (42.9 %)	7 (36.8 %)
III	0 (0 %)	0 (0 %)	1 (5.9 %)	0 (0 %)	0 (0 %)	1 (14.3 %)	1 (5.3 %)
IV	0 (0 %)	0 (0 %)	0 (0 %)	0 (0 %)	0 (0 %)	0 (0 %)	0 (0 %)
Therapy^b^							
Digital	0 (0 %)	0 (0 %)	0 (0 %)	3 (3.3 %)	0 (0 %)	2 (28.6 %)	5 (26.3 %)
ACEi	3 (37.5 %)^h^	4 (30.8 %)^h^	14 (82.4 %)	7 (77.8 %)	0 (0 %)	3 (42.9 %)	15 (78.9 %)
Bblock	1 (12.5 %)	1 (7.7 %)	12 (70.6 %)	9 (100 %)	2 (3.4 %)	6 (85.7 %)	19 (100 %)
ARB	0 (0 %)	3 (23.1 %)^i^	2 (11.8 %)	2 (22.2 %)	0 (0 %)	3 (42.9 %)	4 (21.1 %)
Ca^++^block	0 (0 %)	1 (7.7 %)	1 (5.9 %)	1 (11.1 %)	0 (0 %)	0 (0 %)	1 (5.3 %)
Diuretics	3 (37.5 %)^j^	2 (18.2 %)^j^	7 (46.7 %)	5 (100 %)	0 (0 %)	2 (40 %)	12 (85.7 %)
Aldost. Ant.	0 (0 %)	0 (0 %)	8 (47.1 %)	6 (66.7 %)	0 (0 %)	5 (71.4 %)	12(63.2 %)
Hemodynamics							
HR (bpm)	63.3 ± 4.1	61.9 ± 11.7^k^	63.7 ± 10.9	69.4 ± 9.7	71.1 ± 11.0	64.6 ± 12.4	78.6 ± 15.4
SAP (mmHg)	132.0 ± 12.1	122.5 ± 12.3	122.4 ± 15.5	111.4 ± 18.0	122.4 ± 11.7	118.0 ± 28.5	121.7 ± 15.6
DAP (mmHg)	79.1 ± 2.7	73.9 ± 12.7	74.0 ± 11.5	70.1 ± 12.6	74.6 ± 12.8	70.6 ± 12.8	79.5 ± 15.0

*BS* body surface, *BMI* body mass index, *SAH* systemic arterial hypertension, *DM* diabetes mellitus, *DLP* dyslipidemia, *FC CHF NYHA* functional class of congestive heart failure, *NYHA* New York Heart Association, *ACEi* inhibitor of angiotensin converting enzyme, *β block* beta blocker; *ARB* angiotensin II receptor blocker, *Ca ++ block* calcium channel blocker; *Aldost Ant* aldosterone antagonist, *HR* heart rate, *SAP* systolic arterial pressure, *DAP* diastolic arterial pressure

^a^Continuous variables expressed as mean ± SD. ^b^Categorical variables expressed as frequency (proportion)

^c^Tukey test, *p* < 0.001, vs. C1

^d^Tukey; Ch1A, Ch1B *p* = 0.03 and *p* = 0.04, vs. C1

^e^Fisher’s exact test; Ch1A and Ch1B, *p* < 0.001, vs. C1

^f^Fisher’s exact test; Ch1A and Ch1B, *p* = 0.03; vs. C1

^g^Fisher’s exact test; Ch1A and Ch1B, *p* < 0.001, vs. C1

^h^Fisher’s exact test; Ch1A and Ch1B, *p* < 0.001, vs. C1

^i^Fisher’s exact test, *p* = 0.04, vs. C1

^j^Fisher’s exact test; Ch1A and Ch1B, *p* = 0.01, vs. C1

^k^Tukey test, *p* < 0.02, vs. C1

**Table 2:** Echocardiographic variables

Variables	Chagas				Controls		
	Ch1A (*n* = 8)	Ch1B (*n* = 13)	Ch2 (*n* = 17)	Ch3 (*n* = 9)	C1 (*n* = 58)	C2 (*n* = 7)	C3 (*n* = 19)
LA (mm)^a^	34.4 ± 2.3	32.2 ± 4.7	40.7 ± 4.1	46.6 ± 6.5	33.6 ± 3.2	37.9 ± 6.8	44.1 ± 5.1
LVDD (mm)^a^	47.4 ± 3.9	47.1 ± 4.2	62.4 ± 5.7	74.9 ± 5.8^c^	46.7 ± 4.5	50.7 ± 7.6	67.0 ± 4.9
LVSD(mm)^a^	31.3 ± 3.9	33.5 ± 4.8	50.5 ± 7.2	66.1 ± 5.7	30.5 ± 3.2	45.9 ± 7.0	59.5 ± 5.1
LVFS (%)^a^	34.3 ± 3.1	33.3 ± 4.9	19.3 ± 6.0	11.2 ± 3.7	35.2 ± 2.6	19.0 ± 9.4	11.2 ± 2.6
LVEDV (ml)^a^	82.5 ± 24.6^d^	74.0 ± 29.8	149.5 ± 37.0	262.0 ± 61.7	108.7 ± 28.2	147.1 ± 67.8	216.4 ± 63.4
LVESV (ml)^a^	29.5 ± 11.8	38.5 ± 15.3	84.6 ± 24.0	191.6 ± 43.8	39.6 ± 13.2	87.0 ± 45.8	167.1 ± 49.7
LVEF (%)^a^	65.0 ± 4.3	61.7 ± 5.5	43.8 ± 6.6	26.6 ± 3.9	64.3 ± 4.6	42.1 ± 6.0	23.0 ± 6.1
Diast. Func.^e^							
Normal	5 (62.5 %)	9 (69.7 %)	1 (5.9 %)	0 (0 %)	54 (93.1 %)	2 (28.6 %)	0 (0 %)
Grade I	3 (37.5 %)^e^	4 (30.8 %)^e^	11 (64.7 %)	5 (55.6 %)	4 (6.9 %)	5 (71.4 %)	8 (42.1 %)
Grade II	0 (0 %)	0 (0 %)	5 (29.4 %)	4 (44.4 %)	0 (0 %)	0 (0 %)	5 (26.3 %)
Grade III	0 (0 %)	0 (0 %)	0 (0 %)	0 (0 %)	0 (0 %)	0 (0 %)	1 (5.3 %)
Grade IV	0 (0 %)	0 (0 %)	0 (0 %)	0 (0 %)	0 (0 %)	0 (0 %)	5 (26.3 %)
E Vel (m/s)^a^	0.73 ± 0.17	0.78 ± 0.15	0.65 ± 0.26	0.79 ± 0.34	0.82 ± 0.16	0.61 ± 0.16	0.76 ± 0.24
E DT (ms)^a^	240.6 ± 37.3^f^	214.5 ± 36.3	274.6 ± 81.3	233.6 ± 60.5	190.8 ± 33.3	238.6 ± 61.8	200.9 ± 107.4
A Vel (m/s)^a^	0.71 ± 0.16^g^	0.69 ± 0.17^g^	0.67 ± 0.27	0.69 ± 0.18	0.48 ± 0.12	0.71 ± 0.19	0.60 ± 0.33
S’ Vel (cm/s)^a^	0.06 ± 0.01^h^	0.05 ± 0.01^h^	0.05 ± 0.01	0.04 ± 0.01	0.08 ± 0.02	0.05 ± 0.01	0.03 ± 0.01
E’ Vel (cm/s)^a^	0.06 ± 0.02^i^	0.08 ± 0.02^i^	0.05 ± 0.01	0.03 ± 0.01	0.11 ± 0.02	0.07 ± 0.01	0.04 ± 0.02
A’ Vel (cm/s)^a^	0.09 ± 0.03	0.08 ± 0.02	0.07 ± 0.02	0.06 ± 0.02	0.09 ± 0.02	0.06 ± 0.01	0.04 ± 0.02
E/E’^a^	12.0 ± 4.3^g^	11.8 ± 5.3^g^	13.4 ± 4.7	23.9 ± 11.1	7.9 ± 1.7	9.8 ± 2.5	23.4 ± 11.0
MR Grade^b^							
Absent/Trivial	6 (75.0 %)	9 (69.2 %)	4 (23.5 %)	0 (0 %)	55 (94.8 %)	2 (28.6 %)	1 (5.3 %)
Mild	2 (25.0 %)^j^	4 (30.8 %)^j^	9 (52.9 %)	3 (33.3 %)	3 (5.2 %)	4 (57.1 %)	13 (68.4 %)
Moderate	0 (0 %)	0 (0 %)	3 (17.6 %)	2 (22.2 %)	0 (0 %)	1 (14.3 %)	4 (21.1 %)
Severe	0 (0 %)	0 (0 %)	1 (5.9 %)	4 (44.4 %)	0 (0 %)	0 (0 %)	1 (5.3 %)

^a^Continuous numeric variables expressed as mean ± SD. ^b^Ordinal variables expressed as number of patients (percentage). LA, left atrium; LVDD, left ventricular diastolic diameter, LVSD, left ventricular systolic diameter; LVFS, left ventricular fractional shortening; LVEDV, end-diastolic volume of the left ventricle; LVESV, end-systolic volume of the left ventricle; LVEF, left ventricular ejection fraction; E Vel, E wave velocity, TD E, deceleration time of E wave; A Vel, A wave velocity; S' Vel, S’ wave velocity; E’ Vel, E’ wave velocity; MR degree, degree of mitral regurgitation

^c^Student *t*-test, *p* = 0.01; vs. C3

^d^Tukey test, *p* = 0.04, vs. C1

^e^Fisher’s exact test; Ch1A and Ch1B, *p* = 0.03; vs. C1

^f^Tukey test, *p* = 0.01, vs. C1

^g^Tukey; Ch1A and Ch1B, *p* < 0.05, vs. C1

^h^Tukey; Ch1A and Ch1B, *p* < 0.01, vs. C1

^i^Tukey; Ch1A and Ch1B, *p* < 0.05, vs. C1

^j^Tukey; Ch1A and Ch1B, *p* = 0.01, vs. C1

### Results of STE

#### Global longitudinal analyzes

The global values of LV mechanics analysis obtained from apical views (longitudinal, A4C and A2C) are described in Table 3. Values of global longitudinal velocity in groups Ch1A and Ch1B were lower than control group (Ch1A 3.33 ± 0.44 cm/s vs C1 4.43 ± 0.78 cm/s, *p* < 0.001, and Ch1B 3.38 ± 0.50 cm/s vs C1, *p* = 0.02). In order to assess a possible interaction of this find with other variables, such as hypertension and diastolic dysfunction, we performed an analysis of covariance (ANCOVA) and multiple linear regression, and no interference was found (hypertension, *p* = 0.52, and diastolic dysfunction, *p* = 0.68).

**Table 3:** Global values of left ventricular mechanical contraction analysis obtained from apical views (longitudinal, A4C and A2C)

Variables	Chagas				Controles		
	Ch1A (*n* = 8)	Ch1B (*n* = 13)	Ch2 (*n* = 17)	Ch3 (*n* = 9)	C1 (*n* = 58)	C2 (*n* = 7)	C3 (*n* = 19)
APLAX							
LVel (cm/s)	3.28 ± 0.52^a^	3.81 ± 0.61	3.24 ± 0.77	3.22 ± 1.02^d^	4.42 ± 0.94	3.45 ± 1.26	2.52 ± 0.70
LS (%)	−21.68 ± 2.73	−21.57 ± 2.58	−13.86 ± 2.83	−9.82 ± 2.96	−20.79 ± 2.45	−14.69 ± 2.13	−7.89 ± 2.92
LSR (1/s)	−1.02 ± 0.36	−1.07 ± 0.36	−0.85 ± 0.23	−0.65 ± 0.11	−1.12 ± 0.22	−0.86 ± 0.18	−0.62 ± 0.18
LD (mm)	9.49 ± 1.70	9.47 ± 1.50	6.75 ± 1.77	6.25 ± 1.86^e^	9.60 ± 1.51	7.46 ± 1.10	4.21 ± 1.65
A4C							
LVel (cm/s)	3.54 ± 0.49^b^	4.17 ± 0.60	3.33 ± 0.77	2.90 ± 1.03	4.54 ± 0.92	3.38 ± 0.73	2.73 ± 0.91
LS (%)	−23.16 ± 2.88	−21.06 ± 2.35	−15.54 ± 2.88	−9.96 ± 2.34	−21.16 ± 2.52	−14.73 ± 2.32	−8.48 ± 3.04
LSR (1/s)	−1.14 ± 0.17	−1.13 ± 0.19	−0.86 ± 0.16	−0.63 ± 0.16	−1.20 ± 0.16	−0.83 ± 0.18	−0.71 ± 0.23
LD (mm)	10.80 ± 2.03	10.39 ± 1.41	8.44 ± 1.48	7.73 ± 2.02^f^	10.75 ± 1.60	8.28 ± 1.54	4.83 ± 2.15
A2C							
LVel (cm/s)	3.19 ± 0.58^c^	3.47 ± 0.50^c^	3.01 ± 0.56	2.86 ± 0.78	4.30 ± 0.85	3.33 ± 0.72	2.54 ± 0.65
LS (%)	−22.42 ± 2.80	−22.36 ± 2.22	−16.02 ± 2.96	−10.31 ± 1.36	−22.28 ± 2.82	−15.63 ± 2.42	−8.32 ± 2.90
LSR (1/s)	−1.05 ± 0.14^g^	−1.17 ± 0.12	−0.83 ± 0.16	−0.69 ± 0.10	−1.21 ± 0.18	−0.84 ± 0.14	−0.64 ± 0.13
LD (mm)	10.42 ± 1.65	10.67 ± 1.60	8.72 ± 1.40	6.46 ± 1.52 ^h^	11.09 ± 1.91	8.41 ± 1.90	4.86 ± 1.53
GLOBAL							
LVel (cm/s)	3.33 ± 0.44^i^	3.81 ± 0.50^i^	3.20 ± 0.63	3.00 ± 0.79	4.43 ± 0.78	3.39 ± 0.77	2.60 ± 0.67
LS (%)	−22.42 ± 2.57	−21.67 ± 1.95	−15.14 ± 2.70	−10.03 ± 1.96	−21.41 ± 2.27	−15.01 ± 1.95	−8.23 ± 2.82
LSR (1/s)	−1.07 ± 0.17	−1.12 ± 0.13	−0.85 ± 0.15	−0.66 ± 0.11	−1.20 ± 0.17	−0.84 ± 0.13	−0.66 ± 0.16
LD (mm)	10.23 ± 1.63	10.17 ± 1.36	7.97 ± 1.32	6.48 ± 1.57^j^	10.48 ± 1.45	8.05 ± 1.30	4.63 ± 1.6

Data expressed as mean ± SD. *APLAX* apical longitudinal axis, *A4C* apical, 4-chambers, *A2C* apical 2-chambers, *LVel* longitudinal velocity, *LS* longitudinal strain, *LSR* longitudinal strain rate, *LD* longitudinal displacement, *GLOBAL* refers to the average values of the 3 incidences (APLAX, A4C and A2C)

^a^Tukey, *p* = 0.02; vs. C1

^b^Tukey, *p* = 0.007; vs. C1

^c^Tukey; Ch1A, *p* = 0.01, and Ch1B, *p* = 0.03; vs. C1

^d^Student’s *t*-test, *p* = 0.04, vs. C3

^e^Student *t*-test, *p* = 0.007; vs. C3

^f^Student *t*-test, *p* = 0.04; vs. C3

^g^Tukey test, *p* = 0.03; vs. C1

^h^Student *t*-test, *p* = 0.02; vs. C3

^i^Tukey; Ch1A, *p* <0.001, and Ch1B, *p* = 0.02; vs. C1

^j^Student *t*-test, *p* = 0.01; vs. C3

Longitudinal displacement (LD) and strain in the group with mild/moderate dysfunction were reduced. However, in the group with severe LV systolic impairment (Ch3), there was a paradoxical increase of these parameters in the chagasic group (Global LD: Ch3 6.48 ± 1.57 mm vs C3 4.63 ± 1.60 mm, *p* = 0.01; Table 3).

#### Global short axis analyzes

The global values obtained from LV short axis (basal, middle and apical) view are shown in Table 4. At apical level, chagasic group with severe LV dysfunction (Ch3) had higher radial displacement in comparison with its control C3 (2.49 ± 0.53 mm vs 1.54 ± 1.18 mm, *p* = 0.04). Worthy to note, we found no significant differences in basal and apical rotations, twist/torsion among groups. Data from torsional mechanics analyzes are displayed in Table 5.

**Table 4:** Global values of ventricular mechanical contraction analysis obtained from left ventricular short axis (basal, middle and apical)

Variables	Chagas				Controls		
	Ch1A (*n* = 8)	Ch1B (*n* = 13)	Ch2 (*n* = 17)	Ch3 (*n* = 9)	C1 (*n* = 58)	C2 (*n* = 7)	C3 (*n* = 19)
SAX – MV							
CS (%)	−21.11 ± 4.57	−18.79 ± 3.47	−13.12 ± 2.67	−9.84 ± 2.52	−18.42 ± 2.96	−13.38 ± 2.28	−8.83 ± 2.81
RS (%)	48.92 ± 16.90	53.89 ± 21.25	34.23 ± 22.37	24.84 ± 11.48	47.50 ± 14.64	26.01 ± 15.36	20.91 ± 13.38
CSR (1/s)	−1.56 ± 0.18	−1.50 ± 0.38	−1.04 ± 0.22	−0.82 ± 0.18	−1.47 ± 0.29	−1.17 ± 0.26	−0.83 ± 0.28
RSR (1/s)	2.00 ± 0.35	2.12 ± 0.58	1.46 ± 0.50	1.44 ± 0.46	2.00 ± 0.44	1.73 ± 0.44	1.50 ± 0.62
RD (mm)	6.93 ± 1.37	6.45 ± 1.14	4.84 ± 1.16	4.39 ± 1.43	6.44 ± 1.03	4.64 ± 0.96	3.67 ± 1.23
Rot (^o^)	−8.03 ± 4.64	−6.15 ± 2.55	−4.84 ± 2.41	−2.50 ± 2.38	−5.86 ± 2.38	−4.66 ± 1.63	−3.35 ± 2.50
RotVel (^o^/s)	−66.66 ± 12.87	−52.61 ± 13.66	−41.13 ± 18.90	−27.85 ± 21.27	−64.91 ± 21.11	−48.07 ± 11.20	−33.33 ± 19.40
SAX – PM							
CS (%)	−20.61 ± 4.43	−18.49 ± 2.38	−12.96 ± 3.52	−9.13 ± 1.67	−19.05 ± 3.52	−12.42 ± 2.30	−8.03 ± 2.10
RS (%)	57.19 ± 20.60	50.00 ± 18.72	37.92 ± 17.35	17.11 ± 11.38	50.63 ± 15.37	28.67 ± 15.21	12.88 ± 8.70
CSR (1/s)	−1.48 ± 0.24	−1.36 ± 0.24	−1.07 ± 0.26	−0.82 ± 0.33	−1.41 ± 0.25	−1.03 ± 0.18	−0.73 ± 0.18
RSR (1/s)	2.00 ± 0.46	1.92 ± 0.59	1.52 ± 0.43	1.26 ± 0.45	1.85 ± 0.45	1.57 ± 0.49	1.23 ± 0.62
RD (mm)	7.14 ± 1.75	6.20 ± 0.76	4.90 ± 1.49	3.47 ± 0.80	6.43 ± 1.06	4.40 ± 0.83	3.14 ± 1.17
SAX – AP							
CS (%)	−27.64 ± 6.06	−25.14 ± 4.06	−17.13 ± 4.04	−9.26 ± 4.96	−26.20 ± 6.41	−15.22 ± 3.55	−7.48 ± 3.25
RS (%)	24.20 ± 14.43	15.44 ± 14.01	16.57 ± 12.70	3.62 ± 10.61	25.92 ± 20.46	18.01 ± 18.51	7.86 ± 6.43
CSR (1/s)	−1.51 ± 0.34	−1.63 ± 0.33	−1.09 ± 0.38	−0.71 ± 0.27	−1.76 ± 0.43	−1.10 ± 0.32	−0.68 ± 0.26
RSR (1/s)	1.32 ± 0.46	1.46 ± 0.64	1.19 ± 0.53	1.13 ± 0.77	1.53 ± 0.79	1.13 ± 0.54	0.87 ± 0.37
RD (mm)	4.89 ± 1.14	4.55 ± 1.18	4.13 ± 1.28	2.49 ± 0.83^a^	5.43 ± 1.38	3.47 ± 0.99	1.54 ± 1.18
Rot (^o^)	12.73 ± 2.95	12.89 ± 3.91	6.47 ± 3.71	3.24 ± 4.51	13.15 ± 4.18	6.05 ± 2.99	3.00 ± 3.26
RotVel (^o^/s)	67.65 ± 15.52	80.34 ± 25.54	44.88 ± 15.64	25.65 ± 35.00	85.82 ± 29.84	47.57 ± 27.36	31.25 ± 20.89

Data expressed as mean ± SD. *SAX-MV* short axis, mitral valve, *SAX-PM* short axis, papillary muscles, *SAX-AP* short axis, apical, *CS* circumferential strain, *RS* radial strain, *CSR* circumferential strain rate, *RSR* radial strain rate, *RD* radial displacement, *Rot* rotation, *Rot Vel* rotational velocity

^a^Student *t*-test, *p* = 0.04, vs. C3

**Table 5:** Results of left ventricular twist and torsion

	Chagas				Controls		
Variables	Ch1A (*n* = 8)	Ch1B (*n* = 13)	Ch2 (*n* = 17)	Ch3 (*n* = 9)	C1 (*n* = 58)	C2 (*n* = 7)	C3 (*n* = 19)
*Twist* (^o^)	20.77 ± 6.56	19.03 ± 3.47	11.31 ± 4.14	5.75 ± 6.24	19.00 ± 4.71	10.71 ± 1.87	6.35 ± 3.79
TwVel (^o^/s)	134.31 ± 21.70	132.95 ± 29.81	86.01 ± 24.04	53.50 ± 54.28	150.73 ± 39.54	95.63 ± 33.81	64.60 ± 26.75
Torsion(^o^/cm)	2.64 ± 0.83	2.37 ± 0.65	1.30 ± 0.51	0.65 ± 0.71	2.28 ± 0.61	0.99 ± 0.55	0.78 ± 0.46
T Vel(^o^/s.cm)	17.14 ± 2.89	16.59 ± 5.45	9.66 ± 2.85	6.32 ± 6.16	18.00 ± 4.70	10.12 ± 5.06	7.37 ± 3.03

Data expressed as mean ± SD. *Tw Vel* twist velocity, *T Vel* torsional velocity

### Segmental analyzes

**Fig. 2:**
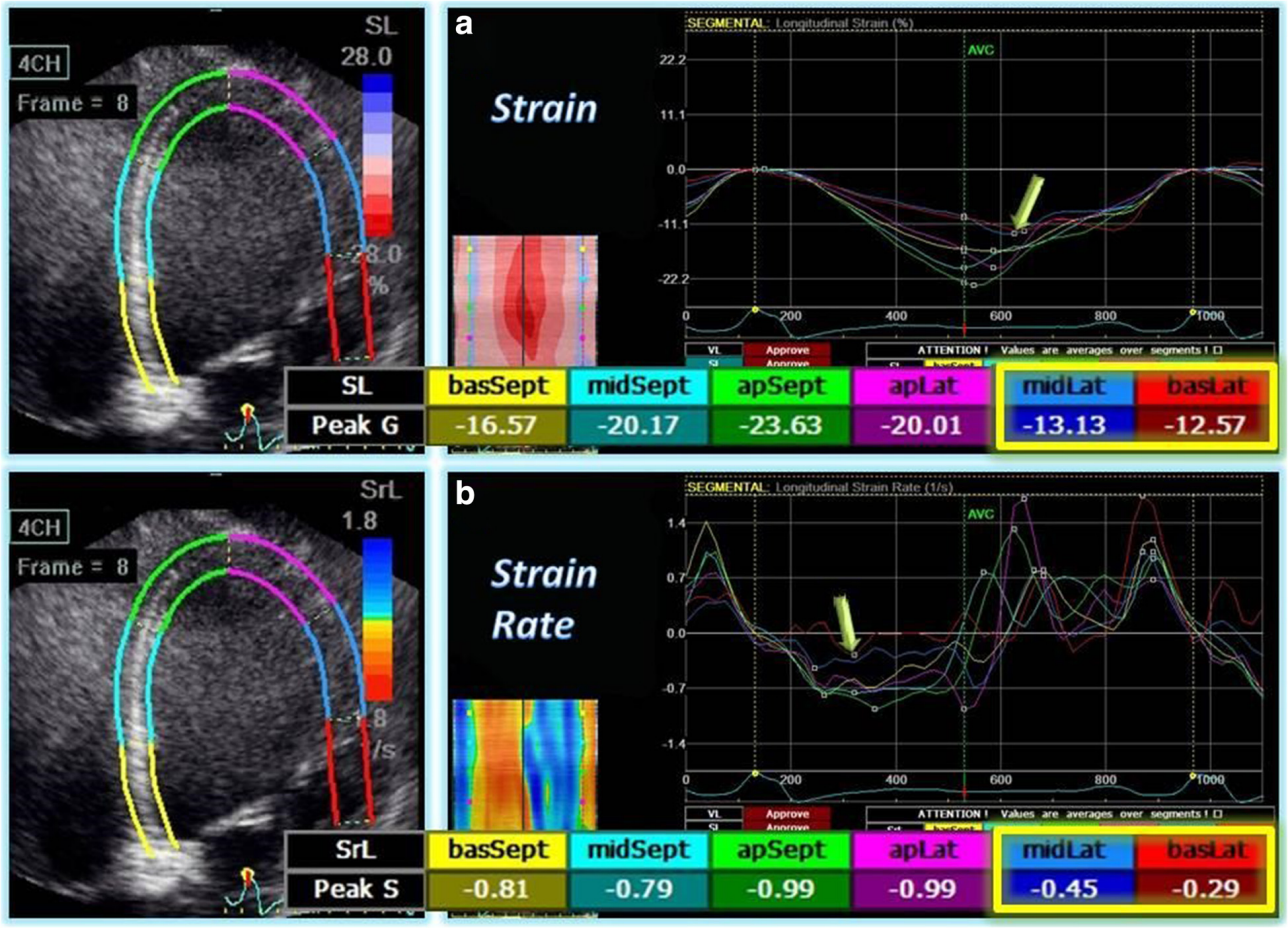
Example of a STE analysis. Patient with CD and normal LVEF. Analyzes of strain (**a**) and strain rate (**b**) in a patient with CD and preserved LVEF, apical four-chamber view. Ventricular myocardium is divided in six color-coded segments and strain/strain rate curves are displayed. Also, small boxes display color m-mode. Note a lack of deformation (worse values; yellow arrows) at the base and mid-segment of lateral wall (red and blue curves, respectively), classically affected by CD

Segmental analyzes were performed and two examples are shown in Tables 6 and 7, highlighting a vicarious pattern of contraction. Segments classically affected by CD, as in inferior and lateral walls, had lower values of radial displacement (RD post: Ch3 2.02 ± 0.90 mm vs C3 3.80 ± 1.92 mm, *p* = 0.03; inf: Ch3 0.92 ± 1.72 mm vs C3 2.22 ± 1.20 mm, *p* = 0.03), and strain/strain rate. On the other hand, an increase of these values was demonstrated in septal and anterior wall segments (RD antsept: Ch3 5.88 ± 2.25 mm vs C3 2.39 ± 1.09 mm, *p* = 0.001; ant: Ch3 5.27 ± 2.49 mm vs C3 3.62 ± 1.50 mm, *p* = 0.04/RS antsept: Ch3 29.15 ± 25.06 % vs C3 8.79 ± 6.80 %, *p* = 0.02). Figure 2 depicts an example of a patient with CD and normal LVEF. STE could identify a lack of deformation (strain and SR) of anterolateral wall basal and medial segments.

**Table 6:** Analysis of mid-left ventricular radial displacement

Varibles	Chagas				Controls		
	Ch1A (*n* = 8)	Ch1B (*n* = 13)	Ch2 (*n* = 17)	Ch3 (*n* = 9)	C1 (*n* = 58)	C2 (*n* = 7)	C3 (*n* = 19)
RD (mm)							
AntSept	8.24 ± 2.13	7.52 ± 1.46	6.60 ± 3.47	5.88 ± 2.25^a^	7.15 ± 1.51	4.13 ± 2.00	2.39 ± 1.09
Ant	7.21 ± 2.26	6.54 ± 1.86	6.62 ± 1.82	5.27 ± 2.49^b^	7.00 ± 1.72	5.72 ± 1.79	3.62 ± 1.50
Lat	6.13 ± 2.21	5.48 ± 1.91	4.31 ± 1.60^c^	3.88 ± 2.69	6.32 ± 1.72	6.00 ± 0.93	4.80 ± 2.00
Post	6.09 ± 2.40	5.14 ± 1.55	3.55 ± 1.51	2.02 ± 0.90^d^	5.80 ± 1.48	4.74 ± 0.78	3.80 ± 1.92
Inf	7.05 ± 2.20	5.90 ± 1.77	3.77 ± 2.28	0.92 ± 1.72^e^	5.96 ± 1.57	3.05 ± 0.54	2.22 ± 1.20
Sept	8.12 ± 1.55^g^	7.25 ± 1.57	5.50 ± 3.12^f^	2.82 ± 3.03	6.55 ± 1.64	2.78 ± 1.06	2.00 ± 1.00

*RD* radial displacement. Numeric continuous variables expressed as mean ± SP

^a^t-Student test; *p* = 0.001; vs C3

^b^t-Student test; *p* = 0.04; vs C3

^c^t-Student test; *p* = 0.02; vs C2

^d^t-Student test; *p* = 0.03; vs C3

^e^t-Student test; *p* = 0.03; vs C3

^f^t-Student test; *p* = 0.004; vs C2

^g^ANOVA/Tukey’s test; *p* = 0.03; vs C1

**Table 7:** Analysis of mid-left ventricular radial strain and strain rate

Variables	Chagas				Controls		
	Ch1A (*n* = 8)	Ch1B (*n* = 13)	Ch2 (*n* = 17)	Ch3 (*n* = 9)	C1 (*n* = 58)	C2 (*n* = 7)	C3 (*n* = 19)
RS (%)							
AntSept	48.38 ± 12.84	48.40 ± 15.41	45.89 ± 24.70	29.15 ± 25.06^a^	44.85 ± 14.55	28.57 ± 15.97	8.79 ± 6.80
Ant	49.92 ± 18.57	50.64 ± 26.51	35.28 ± 18.42	18.43 ± 14.55	43.75 ± 14.18	30.06 ± 17.27	8.39 ± 7.80
Lat	58.15 ± 25.71	55.33 ± 33.18	30.87 ± 18.68	12.52 ± 11.90	49.90 ± 17.34	30.94 ± 22.50	13.14 ± 12.50
Post	65.07 ± 30.01	54.06 ± 27.20	32.78 ± 19.12	7.49 ± 8.54	55.53 ± 20.00	29.66 ± 22.76	17.02 ± 14.11
Inf	63.98 ± 26.45	48.18 ± 18.74	37.84 ± 18.37	11.09 ± 15.96	56.98 ± 19.69	26.82 ± 18.03	16.58 ± 12.16
Sept	57.64 ± 17.73	45.75 ± 17.14	44.86 ± 20.87^b^	23.98 ± 19.59	53.69 ± 18.42	25.95 ± 11.45	13.36 ± 8.83
RSR (1/s)							
AntSept	1.78 ± 0.34	1.87 ± 0.83	1.35 ± 0.62	1.35 ± 0.59	1.69 ± 0.43	1.70 ± 1.20	1.04 ± 0.86
Ant	1.81 ± 0.47	1.94 ± 0.85	1.53 ± 0.45	1.44 ± 0.48	1.75 ± 0.47	1.75 ± 0.68	1.24 ± 1.00
Lat	2.11 ± 0.59	2.04 ± 0.69	1.52 ± 0.45	1.41 ± 0.77	1.88 ± 0.49	1.65 ± 0.43	1.47 ± 0.84
Post	2.24 ± 0.68	1.96 ± 0.59	1.45 ± 0.55	1.08 ± 0.48	1.96 ± 0.51	1.50 ± 0.40	1.28 ± 0.55
Inf	2.12 ± 0.66	1.91 ± 0.51	1.50 ± 0.82	0.93 ± 0.31	1.92 ± 0.50	1.41 ± 0.24	1.22 ± 0.63
Sept	1.91 ± 0.49	1.89 ± 0.77	1.64 ± 0.58	1.33 ± 0.61	1.80 ± 0.46	1.42 ± 0.68	1.15 ± 0.58

Continuos variables expressed as mean ± SD

*RS* radial strain, *RSR* radial strain rate

^a^t-Student test; *p* = 0.02; vs C3

^b^t-Student test; *p* = 0.04; vs C2

### Intraobserver and interobserver variabilities

Interobserver and intraobserver variabilities for longitudinal parameters were 6 %, and 5 %, respectively, with lower variability for longitudinal strain (3 and 4 %).

For the variables obtained from short axis view, including twist and torsion, interobserver variability was 23 %. Circumferential strain rate had the lowest variability (11 %) and torsion the highest (38 %). Intraobserver variability was 19 %, with radial strain rate the lowest value (8 %) and basal rotation the highest (32 %).

## Discussion

This is the first study to fully assess myocardial contraction mechanics in CD throughout its evolution. Although there are a large number of studies using general echocardiography in CD, a recent search using major scientific databases disclosed only two studies that used this relative novel echocardiographic tool (STE) and were similar to ours, conducted by García-Álvarez et al. [25] and Barbosa et al. [26]. However, these study did not include as many variables as we did in our work. Additionally, we included all spectrum of cardiac involvement, from indeterminate form of CD to different stages of LV dysfunction.

Groups in our study were relatively homogeneous, with a few differences in demographics, clinical and echocardiographic parameters. To note, our patients with CD and normal systolic function (Ch1A and Ch1B) were older than our control healthy volunteers (C1), with a higher frequency of hypertension and use of inhibitor of angiotensin converting enzyme. Hypertension, when present in this study was in a mild stage. It is noteworthy that we observed no significant difference on the blood pressure at inclusion examination.

Chagasic patients in the chronic indeterminate form (Ch1A group) had lower longitudinal velocities with statistically significant difference compared to its control group (C1), even after statistical adjusts for hypertension and diastolic dysfunction. This is a very interesting finding and may indicate an incipient myocardial involvement by CD.

A comprehensive and detailed analysis of LV mechanics aimed in our study made it possible to describe the pattern of myocardial contraction in CD. This was evident in the group with severe LV systolic impairment. Segments classically affected by CD, as in inferior and lateral walls, had lower values of radial displacement and deformation (strain/SR). A predilection of CD to affect inferior and inferolateral walls was also demonstrated by Barbosa et al. in their study STE in asymptomatic patients with CD with normal LV systolic function [26]. This author demonstrated lower longitudinal and radial strain in these walls. On the other hand, an increase of these values was demonstrated in septal and anterior wall segments. Although it can be found in more uncommon non-ischemic CMPs (for example: amyloidosis), this feature provide a great help in determining a chagasic etiology of a CMP in locations where CD is very prevalent.

Physiopathogenesis of CD is still not fully understood. Evidences point that a predominant affection of inferior, lateral and apical left ventricular segments may reside on the fact that there is a significant microcirculatory disarrangement on peripheral vascular beds throughout chagasic CMP evolution. In these “watershed zones” located in these myocardial segments, there would be focal ischemia, perpetuating a chronic inflammation, leading to fibrosis [2, 27]. Classical histologic studies demonstrated focal myocardial involvement with localized fibrosis, which differs, for example, from a diffuse CMP [28, 29]. Moreover, these studies described localized foci of myocardial hypertrophy in chagasic CMP, a compensatory mechanism among segments, representing a vicarious pattern of CD. Rochitte et al. and Regueiro et al. used cardiac magnetic resonance with administration of gadolinium in CD [30, 31]. These studies demonstrated evidence of fibrosis in individuals in indeterminate form of CD, increasing in cases of LV dilation and complex ventricular arrhythmia.

García-Álvarez et al. described lower values of radial strain in the middle segment of inferior wall and found differences in LV twist in CD indeterminate form compared to its control group (Ind, 8.7° vs. control, 12.8°; *p* = 0.04). All these findings were not confirmed in our study. Noteworthy is the low values of twist found by this author, mainly due to lower apical rotation (Ind, +5.2° vs. control, +7.6°) [25]. Comparing study populations, based on demographics, we had an older group of patients in the indeterminate form of CD. As shown by van Dalen et al., Takeuchi et al. and Zhou et al., there is a tendency to increase in LV twist with aging [32–34].

### Study limitations

Small number of patients with CD allocated to each subgroup was a limitation of our study. However, it is noteworthy that we performed a segment-to-segment analysis which involved a large number of myocardial segments in each subgroup and this enabled our study to disclose the significant difference among variables.

The subjectivity of echocardiography can bring biases of quantification. This is exemplified when referring to the “cutting” level of the LV in its apical short axis, which has no anatomical landmark. So, small variations in the level of image acquisition can lead to distorted values. Our new criterion created for this study – an identification of at least a tendency to rotation of the apex – tried to preclude this issue.

Despite STE method is validated in the literature, it is still an evolving technique, and improvements, such as the accuracy of the tracking, are still needed.

## Conclusions

Left ventricular systolic mechanics analysis disclosed a vicarious pattern of contraction in the course of chagasic CMP evolution. CD with LV mild/moderate systolic impairment had a global reduction in all systolic parameters. However, a major reduction of displacement and strain into inferior and posterior walls had an opposite paradoxical increase of these parameters into septal and anterior segments. This may help defining a non-ischemicMP etiology, providing robust elements to point to a chagasic cause where this disease is prevalent. Additionally, patients in the indeterminate form of CD presented with lower longitudinal velocities, which may indicate an incipient myocardial injury.

## Abbreviations

CD, Chagas disease; CMP, cardiomyopathy; LVEF, left ventricular ejection fraction; STE, speckle tracking echocardiography; A4C, apical four chambers; A2C, apical two chambers; A3C; apical theee chambers or long-axis; LD, longitudinal displacement; LS, longitudinal strain; LSR, longitudional strain rate; RD, radial displacement; RS; radial strain; RSR, radial strain rate; CS, circumferential strain; CSR, circumferential strain rate
